# Temporal and demographic variation in partial migration of the North Atlantic right whale

**DOI:** 10.1038/s41598-018-36723-3

**Published:** 2019-01-23

**Authors:** Timothy A. Gowan, Joel G. Ortega-Ortiz, Jeffrey A. Hostetler, Philip K. Hamilton, Amy R. Knowlton, Katharine A. Jackson, R. Clay George, Cynthia R. Taylor, Patricia J. Naessig

**Affiliations:** 1 Fish and Wildlife Research Institute, Florida Fish and Wildlife Conservation Commission, St. Petersburg, Florida 33701 USA; 2 Department of Wildlife Ecology and Conservation, University of Florida, Gainesville, Florida 32611 USA; 3 Cooperative Institute for Marine and Atmospheric Studies, Rosenstiel School of Marine and Atmospheric Science, University of Miami, Miami, Florida 33149 USA; 4 Anderson Cabot Center for Ocean Life, New England Aquarium, Boston, Massachusetts, 02110 USA; 5 Georgia Department of Natural Resources, Wildlife Conservation Section, Brunswick, Georgia 31520 USA; 6 Sea to Shore Alliance, Sarasota, Florida 34233 USA

## Abstract

Animal movement plays a fundamental role in the ecology of migratory species, and understanding migration patterns is required for effective management. To evaluate intrinsic and environmental factors associated with probabilities of endangered North Atlantic right whales *Eubalaena glacialis* migrating to a wintering ground off the southeastern United States (SEUS), we applied a multistate temporary emigration capture-recapture model to 22 years of photo-identification data. Migration probabilities for juveniles were generally higher yet more variable than those for adults, and non-calving adult females were the least likely group to migrate. The highest migration probabilities for juveniles and adult males coincided with years of relatively high calving rates, following years of higher prey availability in a fall feeding ground. Right whale migration to the SEUS can be classified as condition-dependent partial migration, which includes skipped breeding partial migration for reproductive females, and is likely influenced by tradeoffs among ecological factors such as reproductive costs and foraging opportunities that vary across individuals and time. The high variability in migration reported in this study provides insight into the ecological drivers of migration but presents challenges to right whale monitoring and conservation strategies.

## Introduction

Migration, or the seasonal movement of individuals between habitats, determines the distribution of animals across space and time and affects processes at many levels, from survival of an individual to ecosystem dynamics^1^. Characterizing patterns of migration is therefore imperative to understanding ecological and evolutionary processes, as well as conservation and management issues. Partial migration, where a proportion of a population stays resident in one habitat and the remainder migrates to another habitat, has been documented across a wide range of taxa, including invertebrates, fish, birds, and mammals^2^. In populations that exhibit partial migration, migratory status may be fixed, with individuals classified as either migrants or non-migrants, or it may be condition-dependent, in which migratory status is plastic and determined by intrinsic (*e.g*., age) or extrinsic (*e.g*., resource availability) states^3^.

Baleen whales are known to migrate from high-latitude, summer feeding areas of high productivity to low-latitude, winter breeding areas where feeding is absent or limited^4^. Hypothesized selective pressures driving this behavior in reproductive females relate to calf survival, through energy budgets^5^ or predator densities^6^, but the reasons why other demographic groups migrate to low-latitude areas where feeding is absent remain less clear. Studies of mysticete populations that deviate from this traditional model of migration, including those that exhibit partial migration and non-migratory behavior, may provide insight into factors that regulate migration and its variability^7^.

**Figure 1:**
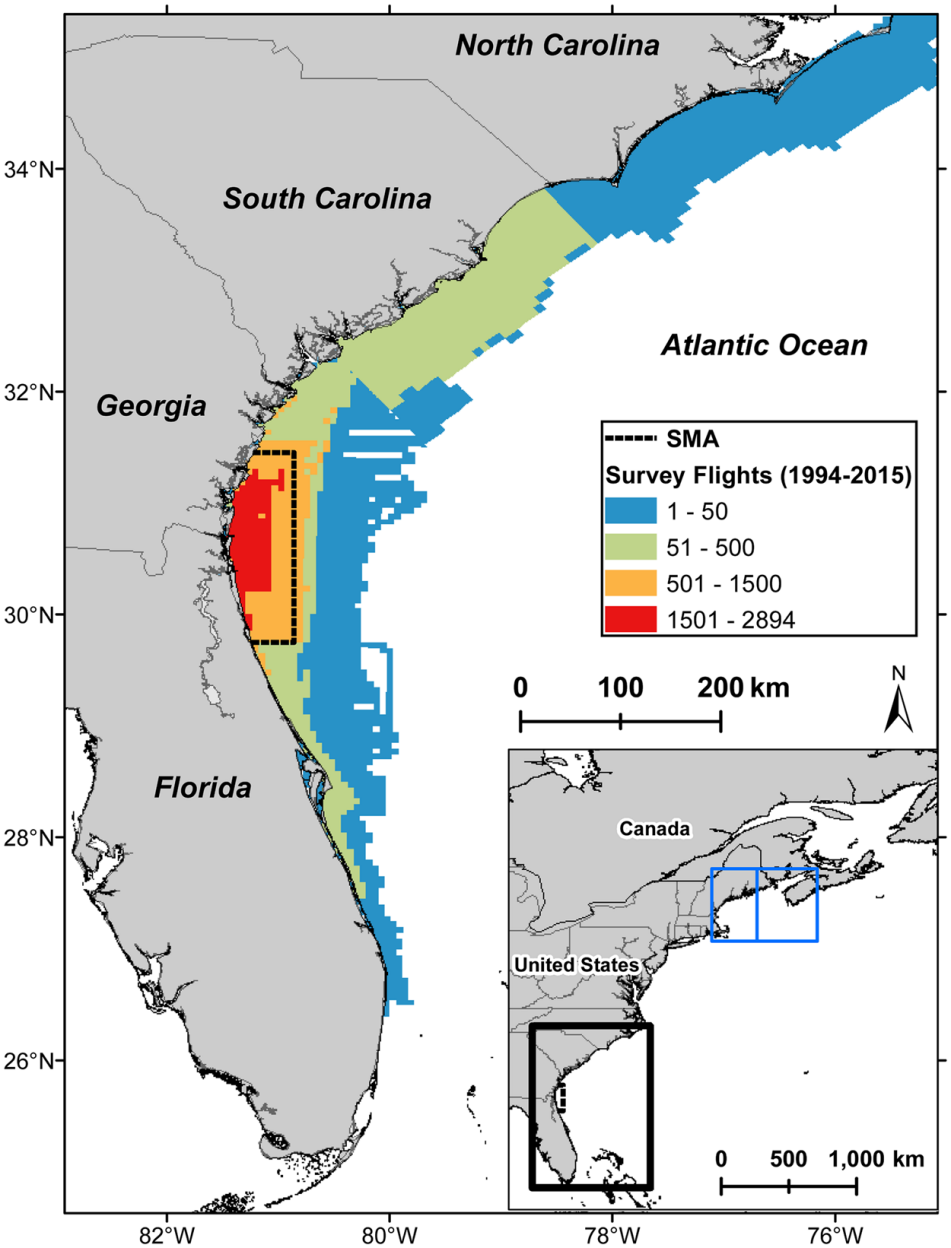
Southeastern U.S. study area. Colors indicate cumulative aerial survey effort during 1994–2015, within and around the right whale seasonal management area (SMA; dashed black line). The Gulf of Maine region used to summarize sea surface temperature and copepod data is shown as a blue line in the inset map, dividing the eastern and western Gulf of Maine.

The endangered North Atlantic right whale *Eubalaena glacialis* (hereafter “right whale”) is widely distributed throughout the western North Atlantic Ocean^8^ but is known to use feeding grounds in and around the Gulf of Maine during spring through fall, and calving grounds off the southeastern United States (SEUS) near Florida and Georgia during the winter^9^ (Fig. 1). The SEUS is commonly referred to as a calving ground, where females migrate to give birth but not mate^9,10^. Previous studies demonstrate that many individuals remain in northern feeding areas during winter^10,11^; however, some right whales representing all demographic groups, including juveniles, adult males, and non-calving females, have been documented in the SEUS during winter^12,13^.

Since 2010, monitoring surveys have detected fewer right whales in several, traditionally high-use habitats^14,15^. Along with changes in survey effort and population size, a leading hypothesis for this decrease in detections in certain foraging habitats is a shift in right whale spatial distribution in response to variation in environmental conditions, including the distribution and availability of food resources. More specifically, bottom-up processes, such as the North Atlantic Oscillation (NAO) index and sea surface temperature (SST), and the abundance of *Calanus finmarchicus* copepods, the primary prey for right whales, have been previously hypothesized to influence right whale energetic budgets, reproductive dynamics, and distribution^16,17^. Long-term capture-recapture data from the photographic identification of individual right whales provide a means to examine patterns of habitat use across demographic groups^11^, across years, and as a function of environmental conditions.

Multistate capture-recapture methods have been used to simultaneously estimate probabilities of survival (*S*), capture (*p*), and transitions (ψ) between states that are potentially relevant to fitness, including reproductive state, physiological state, and geographic location^18,19^. In standard capture-recapture models, an individual has a non-negligible recapture probability during each sampling period unless it has died or permanently emigrated from the population^20^. Extensions to capture-recapture models have been developed to allow for temporary emigration, in which some individuals are absent from the survey area and thus unavailable for detection during one or more sampling periods. A common approach is the use of a multistate model where individuals may transition between an observable state and an unobservable state; individuals in the sampling area are in an observable state and thus available for capture, and moving to an unobservable state is equivalent to temporary emigration^20^. Temporary emigration results in heterogeneity in recapture probabilities and in some cases can bias estimates of survival (*e.g*., when temporary emigration probability depends on the previous state and is thus Markovian)^20–22^. Models that account for temporary emigration therefore have been used to improve survival estimates, but temporary emigration can itself be a parameter of ecological interest^20,23^.

**Figure 2:**
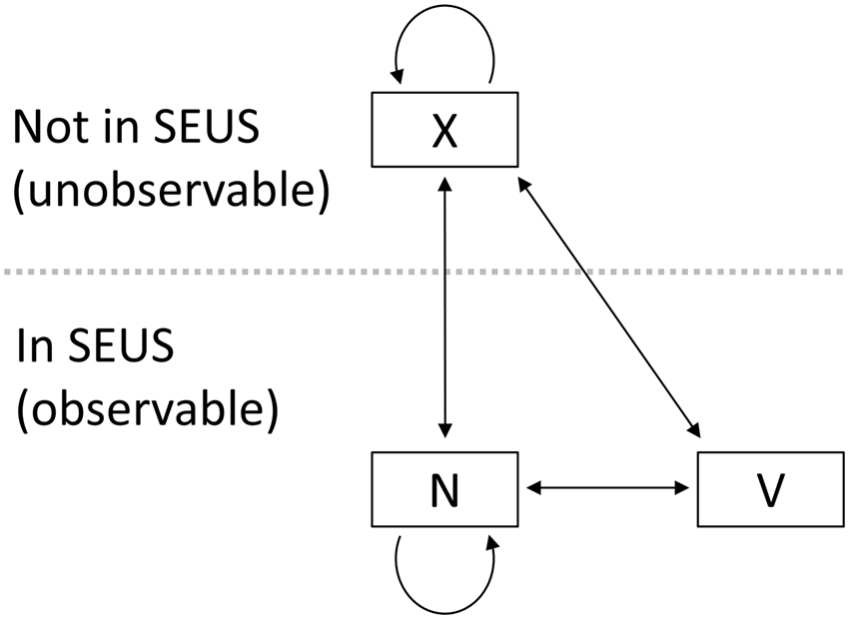
Allowable transitions for the multistate right whale model. States are defined as N = non-breeder, V = calving female, and X = unobservable. Males and individuals of unknown sex are not permitted to transition to or from the calving female state. Females are not permitted to calve in consecutive years. Transitions to an observable state (N, V) represent migration to the southeastern U.S. (SEUS) study area.

In this study, using a robust sampling design with relaxed closure assumptions^24,25^, we applied a multistate temporary emigration model to estimate the probability of right whales migrating to their winter calving grounds in the SEUS. Individuals were considered to be in one of three states each winter season: breeder (calving females) in the SEUS (V), non-breeder (males and non-calving females) in the SEUS (N), or not in the SEUS (unobservable; X) (Fig. 2). Data for this study were limited to aerial survey sightings in the SEUS right whale Seasonal Management Area (SMA; Fig. 1). Whales present in the SEUS in a given winter were considered to be in an observable state that year, and transitions to an observable state were used to infer migration to the SEUS. Migration probabilities were modeled across demographic groups, years, and environmental covariates.

This model framework was used to test the following hypotheses: (1) right whales exhibit condition-dependent partial migration, and thus individuals documented in the SEUS do not migrate there every winter; (2) energetic budgets influence migration probability, and thus migration will depend on an individual’s migratory or reproductive state in the previous year (i.e., it is a Markovian process); (3) males and females have different migration probabilities due to different reproductive requirements and pressures; (4) juveniles and adults have different migration probabilities due to different thermoregulatory, predation, reproductive, or intraspecific competition pressures; (5) migration probabilities are higher during colder winters; and (6) migration probabilities are higher following periods of high prey availability. Understanding the factors that affect migration, and thus the distribution of a population and its variability across years and demographic groups, can improve the effectiveness of monitoring programs and conservation actions by informing the timing and location of surveys and protection zones.

## Results

A total of 455 individual, non-calf right whales (226 females, 211 males, 18 of unknown sex) were observed and identified from 7,565 sighting records by Early Warning System (EWS) aerial surveys in the SEUS SMA during 1994–2015. This represents 91% of individuals observed south of the Virginia/North Carolina border, and 73% of individuals observed range-wide, from all sighting sources (boat, land, aerial) during the same period. The number of individuals observed in the SMA by EWS aerial surveys ranged from 10 whales in 1999 to 196 whales in 2009 (Supplementary Fig. S1 and Table S4). Using sighting data from all regions, 150 females were known to have calved a cumulative 381 times during 1994–2015. For 57 (15%) of these cases, the female was either not seen at all (n = 45) in the SMA by EWS aerial surveys that winter or seen but without a calf (n = 12; still classified as a calving female in this study).

**Figure 3:**
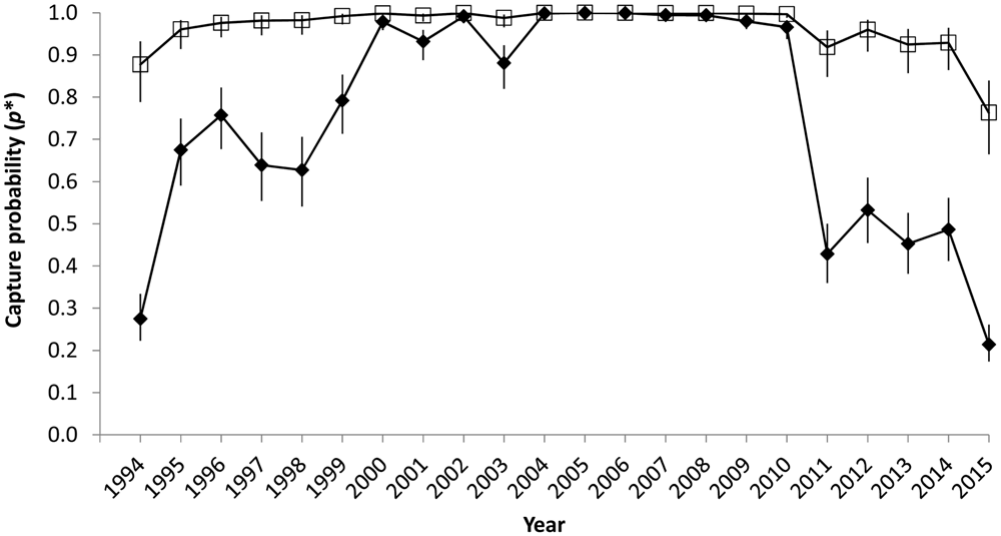
Estimates of winter capture probability (with 95% confidence intervals) for non-breeder (diamonds) and calving female (open squares) right whales in the southeastern U.S. seasonal management area. Estimates are derived from model 8.time (Supplementary Table S5).

Goodness of fit tests indicated no evidence of lack of fit for our capture-recapture models (*ĉ* < 1.19, *P* > 0.29 for all tests). In the model best supported by Akaike’s Information Criterion corrected for small sample sizes (AICc)^26^, *S* did not differ between juveniles and adults, *p* had interaction effects of state and survey effort, and ψ had full time variation (i.e., different transition probabilities for each year of the study) with temporal patterns shared across some demographic groups (model 8.time, Supplementary Table S5). In this model, annual *S* was estimated to be 0.968 (95% confidence limits: 0.959–0.975). Calving females had higher *p* than non-breeders in the SEUS, and survey effort positively influenced *p* for non-breeders but was non-significant (confidence limits for effect included 0) for calving females. The probability of detecting an individual at least once in a given winter (${p}_{t,j}^{\ast }=1-[1-{p}_{t,1,j}]\ast [1-{p}_{t,2,j}]$) was close to 1 in most years for calving females in the SMA but was lower and more variable for non-breeders (Fig. 3).

For ψ, models without temporary emigration and where partial migration was not condition-dependent (demographic model 3) consistently received the least support. Models with no differences between juveniles and adults (demographic model 4) or where temporary emigration was not Markovian (demographic model 2) also received little support (Supplementary Table S5). Demographic model 7 consistently had more support than demographic model 6, indicating a sex effect even after controlling for female reproductive state. For temporal variation in ψ, models with full time variation generally had the most support, while models with no variation across time had the least support. Overall, the top four models included full time variation in ψ with time effects that were shared across some or all demographic groups (Supplementary Table S5).

**Figure 4:**
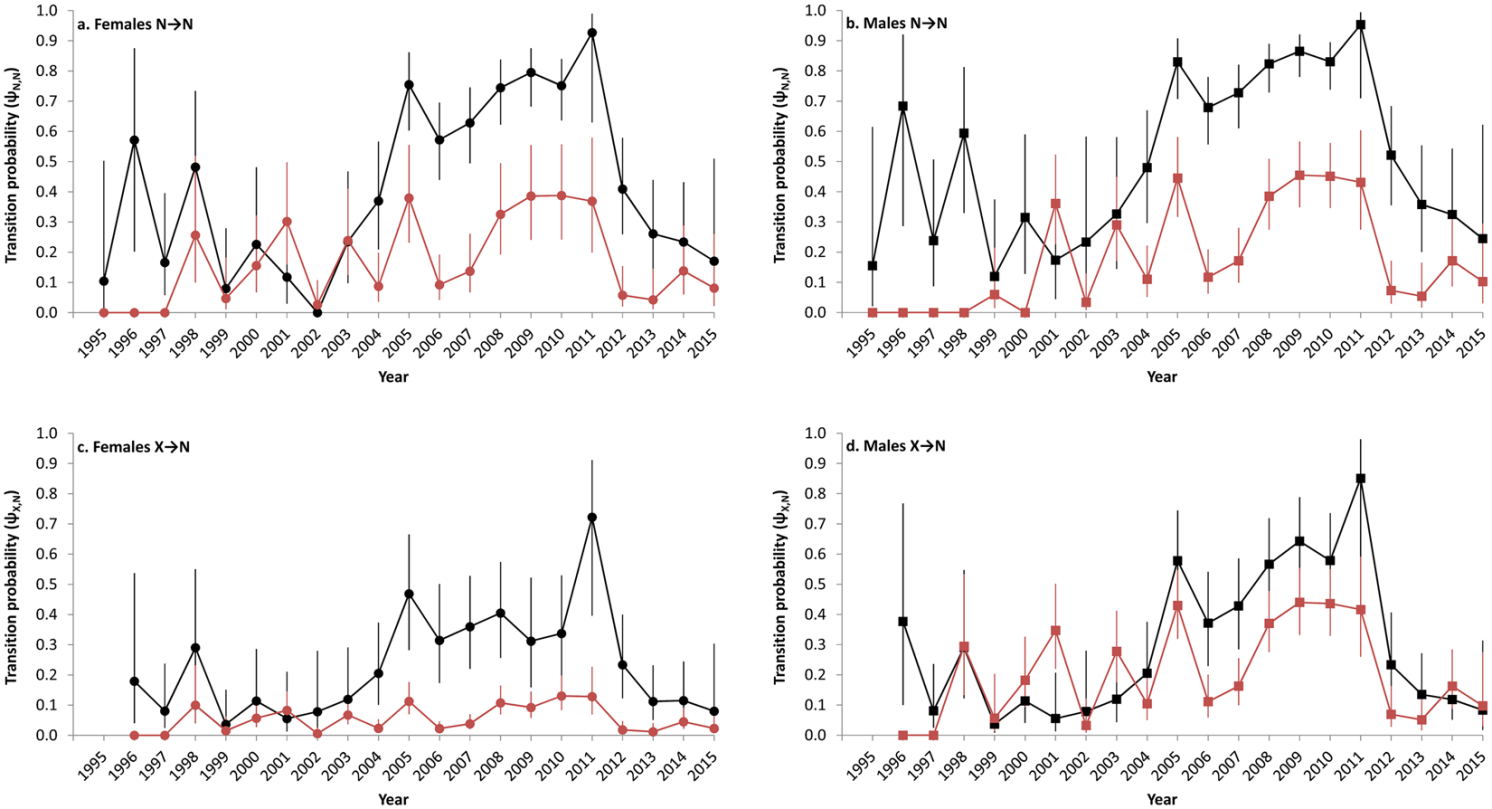
Estimates of transition probabilities (with 95% confidence intervals) for (**a**,**c**) female and (**b**,**d**) male right whales, from (**a**,**b**) non-breeder in the southeastern U.S. (N) to non-breeder in the southeastern U.S. (N) and from (**c**,**d**) not in the southeastern U.S. (X) to non-breeder in the southeastern U.S. (N); black = juveniles, red = adults. Estimates are derived from model 8.time (Supplementary Table S5).

**Figure 5:**
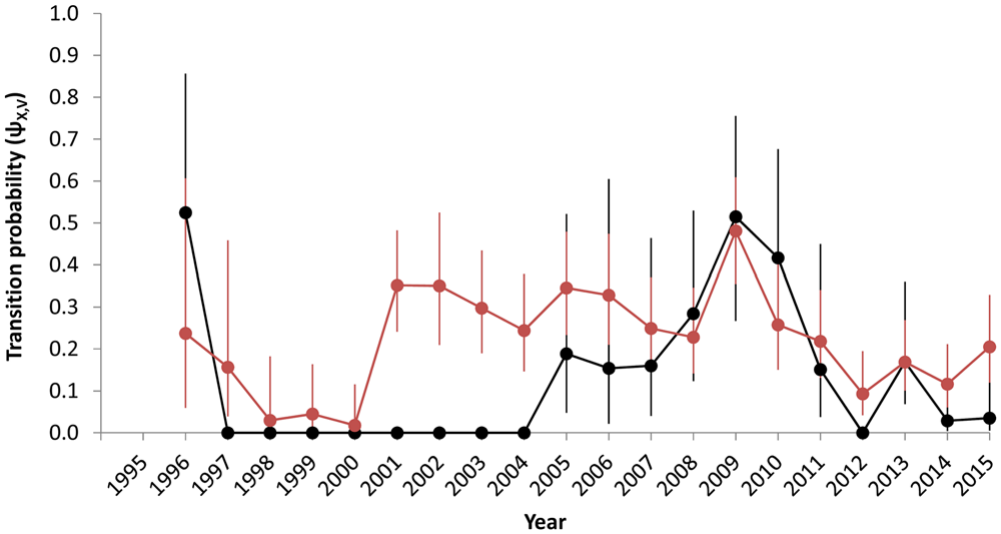
Estimates of transition probabilities (with 95% confidence intervals) from not in the southeastern U.S. (X) to the calving state (V) for female right whales; black = juveniles, red = adults. Estimates are derived from model 8.time (Supplementary Table S5).

In the top model overall (model 8.time, Supplementary Table S5), migration probabilities for non-breeding adults were generally lower and less variable than those for juveniles (Fig. 4). Migration probabilities for juveniles increased from 2002 to 2005, remained elevated through 2011, then steadily declined through 2015. Juveniles and adult females were generally more likely to migrate as non-breeders if they migrated as non-breeders in the previous year (Fig. 4a,b) than if they did not migrate in the previous year (Fig. 4c,d). The probability of a female migrating to calve one year after it migrated as a non-breeder was close to 0 (mean ψ_*t*,N,V_ = 0.01 across all years for both juveniles and adults); indeed, there were only two cases in our dataset where a female was seen in the SEUS the year immediately prior to calving. Similarly, females were unlikely to migrate to the SEUS one year after calving (mean ψ_*t*,V,N_ = 0.07). Calving rates for adult females were highest in 2001–2010, while less than 23% of adult females migrated to the SEUS to calve in 1997–2000 and 2011–2015 (Fig. 5). Calving rate estimates for females younger than nine years old were generally lower and less precise than those for adults but highest in 2005–2010.

Of the models that included environmental covariates for ψ, a fall *C. finmarchicus* abundance index for the eastern Gulf of Maine averaged over the preceding two years tended to be the best predictor of right whale migration to the SEUS, while a lagged NAO index had the least support as a covariate (Supplementary Table S5). The environmental covariate models generally predicted higher probabilities of right whale migration to the SEUS following years of high *C. finmarchicus* abundance and low SST, and during winters with negative NAO indices. In the top environmental covariate model (model 1.ECAL_2avg, Supplementary Table S5), the averaged fall *C. finmarchicus* index for the eastern Gulf of Maine was positively associated with: juvenile and adult females transitioning from the unobservable to calving state; juvenile females, juvenile males, and adult males transitioning from the unobservable state to non-breeder in the SEUS; and juvenile males migrating to the SEUS in consecutive years. However, this index was negatively associated with adult females migrating to the SEUS as non-breeders in consecutive years (Table 1).

**Table 1:** Effect of fall *C. finmarchicus* index in the eastern Gulf of Maine averaged over the preceding two years on right whale state transition probabilities.

Transition	Direction	Estimate	LCL	UCL
X:V juvenile female	+	2.2	0.8	3.5
N:V juvenile female	ns	−1.4	−9.9	7.1
X:V adult female	+	1.4	0.9	2.0
N:V adult female	ns	−1.8	−9.0	5.3
X:N juvenile female	+	1.6	0.1	3.0
N:N juvenile female	ns	0.5	−0.5	1.6
V:N juvenile female	ns	4.5	−1.3	10.2
X:N adult female	ns	0.9	−0.2	2.0
N:N adult female	−	−2.7	−4.8	−0.7
V:N adult female	ns	1.6	−0.5	3.7
X:N juvenile male	+	3.4	1.8	5.1
N:N juvenile male	+	2.1	1.0	3.2
X:N adult male	+	1.4	0.7	2.1
N:N adult male	ns	−0.1	−1.2	1.1

Direction indicates a positive (+), negative (−), or non-significant (ns; confidence limits include 0) relationship. Coefficient estimates are given with lower (LCL) and upper (UCL) 95% confidence limits from model 1.ECAL_2avg (Supplementary Table S5). States are N = non-breeder in the southeastern U.S. (SEUS), V = calving female in the SEUS, and X = not in the SEUS.

## Discussion

By simultaneously modeling movement, survival, and capture probabilities, and by tracking the age and demography of individuals, we were able to quantify variation in habitat use for right whales and characterize its relationship with environmental covariates while accounting for changes in survey effort and population structure. For example, the increased number of juveniles sighted in the SEUS from 2005–2011 (Supplementary Table S4) was due in part to increases in survey effort (Fig. 3) and the number of juveniles in the population (Fig. 5), but also reflected real changes in migratory behavior (Fig. 4). Our results support the hypothesis that all individual right whales in the population have the potential to migrate to the SEUS each winter but that this migration is condition-dependent. Although well-established as a calving ground, our results show that the SEUS is also an important wintering ground used by all other demographic groups. However, use of this habitat varies substantially across demographic groups and years.

Models with the most support showed some level of synchrony across demographic groups in temporal patterns of migration. In particular, the highest migration probabilities for juveniles and adult males coincided with years of high calving rates (Figs 4 and 5). Such synchrony can result from environmental conditions in feeding areas^27^. The support for models with *C. finmarchicus* abundance as a predictor of right whale migration (relative to models with other environmental covariates or no temporal variation) provides evidence that increased prey availability, and its presumed influence on whale energetic budgets and physiological condition, increase the probability of migration to the SEUS in succeeding winters. Similarly, previous studies of cyprinid fish have shown that individuals in poor physiological condition are more likely to remain resident in feeding grounds than incur the energetic costs of migration^1,28^. However, models with full time variation in ψ had substantially more support than models with environmental covariates. This could be due to the coarse resolution (spatially or temporally) of the covariates used, which may be insufficient to fully capture the environmental conditions experienced by right whales. Also, these factors may influence migration in concert rather than in isolation, and they may interact with other factors not considered in this study, such as changes in health^29^ or energetic impacts from entanglements^30^.

We found higher migration probabilities for juveniles than adults. Although smaller individuals have a higher per-gram metabolic rate, larger individuals are expected to have higher total energetic requirements. Jahn, *et al*.^31^ hypothesized that energetic demands are determinants of partial migration, and large individuals may be more likely to over-winter in feeding grounds to meet these demands, with all individuals more likely to over-winter in feeding grounds following years of low prey availability. Additionally, a competitive release hypothesis predicts that subordinates (often the younger or smaller individuals of a population) are more likely to migrate in response to intraspecific competition for resources^2^. It has also been proposed that smaller whales may be less tolerant to cold^5,32^ (but see Ryg, *et al*.^33^) or more vulnerable to predation^6,34^ at high latitudes, although indices of winter temperature had minimal support in our results and we were not able to evaluate inter-annual variation in predation risk.

Males were more likely to use the SEUS than non-calving females, and this pattern was more apparent in adults and for individuals that had not migrated in the previous year (Fig. 4c,d). Craig and Herman^35^ also reported higher sighting rates across years for male than for female humpback whales (*Megaptera novaeangliae*) in wintering grounds off Hawaii, and suggested that this finding may be related to higher energetic costs of reproduction incurred by females (approximately one year of gestation and one year of nursing) compared to males. Therefore, females may be less likely to migrate and forego feeding except in years when they calve. The lack of use of the SEUS by female right whales in the years immediately preceding and following calving suggests that females may overwinter in feeding areas these years to increase energy stores for future reproduction. Periods of low prey availability may increase the number of years between successful calving events^16^ and their associated migrations. Skipped breeding partial migration, a behavior where females do not migrate in years they do not reproduce, has been documented in many taxa where individuals can increase their lifetime reproductive success by skipping breeding and its associated migrations in some years^36^. Individuals are expected to skip breeding more frequently in populations with low mortality, high reproductive costs, and high environmental stochasticity^36^. The pattern of low and variable calving rates observed in right whales is likely related to a strategy of skipped breeding partial migration, long life expectancy, and environmental variability.

Although we identified intrinsic and extrinsic factors associated with variability in right whale migration, the potential fitness benefits for right whales using the SEUS are not clear. Previous researchers have hypothesized that cow-calf pairs incur fitness gains from using low-latitude wintering grounds^5,6^, and the same benefits could be gained by other demographic groups. While feeding and mating opportunities in the SEUS are unlikely^10^, low-latitude wintering grounds may provide juvenile and adult whales with similar thermoregulatory or predator-avoidance benefits as calves. Individuals may also benefit from social behaviors in these wintering grounds, such as mating practice or bond development^37^, which is supported by the frequent observations of social aggregations in the SEUS^38^. Some of these potential benefits (*e.g*., predator avoidance and social development) may be density-dependent, contributing to the observed synchrony in migration probability^13^. Continued analyses of capture-recapture and parentage data could be used to compare fitness between non-migrants and migrants to better understand the selective pressures of right whale migration.

For right whales, the realized ecological trade-off between migrating to the SEUS and overwintering in northern habitats will likely change with variation in environmental conditions across time and variation in intrinsic states across individuals. Migratory flexibility at an individual level can result in highly variable temporal patterns of migration at the population level^39^, as we have documented for right whales. Furthermore, projected changes in oceanographic conditions and *C. finmarchicus* availability^40^, or factors related to changes in right whale population size (*e.g*., the ability to find mates), could result in future right whale migration patterns that differ significantly from historical patterns. Instead of displaying high fidelity to seasonal habitats, right whale movements are complex and variable, and therefore present challenges to monitoring and conservation strategies.

While estimating survival probability was not a primary objective of this study, our results (*S* = 0.968) are generally consistent with other, recent survival estimates for this population (*S* = 0.97–0.99 for non-calves^41,42^). We did not detect differences in survival between juveniles and adults, but calves were excluded from our analysis and we caution that our survival estimates may be biased low, especially for adults, since the low migration probabilities in recent years increase our uncertainty about the fate of individuals^43^. Estimates would likely be improved by incorporating carcass recovery data and sightings from other areas, but we would still advocate the use of temporary emigration models to estimate survival for right whales, as they can account for processes such as reproductive state and differential use of sampling areas that cause heterogeneity in capture probabilities^44^. Additionally, our extension of robust design models permits the incorporation of multiple sightings of an individual within a sampling period to improve parameter estimates, while relaxing typical closure assumptions.

Our results also provide insights on variability in reproductive rates, documenting relatively higher calving rates in 2001–2011 and younger age at first reproduction in 2005–2011. We believe probabilities of transition between reproductive states are a more useful metric for reproductive rates than the commonly-used observed inter-birth interval^45,46^, which is biased by imperfect detection, limited to individuals that calved more than once during the duration of the study, and a lagging indicator (*e.g*. inter-birth intervals in 2001 were likely high even though calving rate was high that year). However, our study will underestimate reproductive rates for females that were incorrectly classified as non-breeders (i.e., whose calf was not detected) in some years and for females that do not always use the SMA in years they calve. Despite high capture probabilities for calving females using the SMA (see also Krzystan, *et al*.^47^), 15% of documented births in the population were not detected in this region (see also Patrician, *et al*.^48^ and McLellan, *et al*.^49^). Survey data from other areas, models that account for uncertainty in reproductive state^50^, or analyses of hormone data^51,52^ are required to provide accurate estimates of reproductive rates for the population.

Monitoring and protective measures in the SEUS are critical to right whale management due to the importance of this habitat as both a calving and wintering ground. Observations in this habitat may serve as an indicator of the population’s overall status. However, the effectiveness of location-based management actions (*e.g*., spatio-temporal fishery closures to mitigate entanglements and vessel speed reduction zones to mitigate ship strikes) may be limited for populations with variable site fidelity, especially if these management boundaries are static across time. Even when an individual migrates, there may be variability in when it arrives or departs^47^ and how far south it travels, which may all be influenced by changing environmental conditions. Such differential migration can result in regional threats having disproportionate effects on different components of a population^53^. As such, populations with variable habitat use may benefit from dynamic management actions that utilize predictive models and real-time observations^54^. A better understanding of right whale distribution and movements, including factors that influence the timing and magnitude of migrations, can help managers target mitigation plans more efficiently in space and time to avoid overly broad impacts to industry and other ocean users. With this goal in mind, continued survey efforts, continued monitoring of environmental data such as the EcoMon plankton surveys^55^, and fine-scale spatio-temporal data on right whale movements such as those from telemetry studies are required to inform and validate predictive models that can be used to better understand ecological drivers of migration and effectively manage the right whale population.

## Methods

### Right whale data

Systematic aerial surveys for right whales have been conducted in the SEUS through the EWS network from approximately December through March since 1994^56^. Surveys consist of daily, weather-dependent flights (targeting a visibility of at least 3.7 km and a Beaufort sea state of 4 or less) from Cessna 337 Skymaster or de Havilland DHC-6 Twin Otter aircraft (see Keller, *et al*.^57^ and Gowan and Ortega-Ortiz^58^ for additional details of survey protocols). Survey teams broke from planned flight paths to circle right whale sightings and collect photographs of whales’ unique patterns of callosities, scarring, and skin pigmentation, which are used to identify individuals and integrated into the North Atlantic Right Whale Consortium Identification Database^12^. Photo-identification records obtained from the Identification Database for this analysis were limited to those from EWS aerial surveys in the right whale SEUS SMA (~9,100 km^2^; Fig. 1) from 1994 to 2015, although sightings outside of this area and timeframe informed age, sex, and reproductive state^59^. This subset of records (representing 67% of all records south of the Virginia/North Carolina border during the same time period) allowed us to make inferences across years within a consistent spatial extent and for which survey methods were comparable and effort could be quantified. A year was defined as November 1 to October 31 to correspond with the winter calving season.

Individuals were assigned sex (female, male, or unknown) based on observations of the genital area, genetics, or close association with a calf^60^. In models with sex effects, whales of unknown sex (4% of individuals in this study) were pooled with males, assuming that females are more likely to be assigned sex due to calving behaviors. Females were further classified as either calving or non-calving each year based on associations with a calf in the SEUS or elsewhere during that year. Age for each individual was tracked across years, and individuals were classified as either juveniles (<nine years old, regardless of their reproductive history) or adults (≥nine years old)^61^. Most individuals in this study (87%) were either seen during their year of birth and were of known age, or had a sighting history of >eight years by 1994 and were classified as adults. Whales of unknown age with a sighting history of <nine years were considered to be one year old during the year that they were first sighted^62^, except those seen as calving females during their first year sighted, which were assumed to be nine years old. Sightings of individuals during the winter they were born (first-year calves) were excluded from this analysis.

### Environmental data

Environmental data were summarized for use as covariates to explain inter-annual variation in right whale migration. Summer Gulf of Maine SST and a concurrent winter NAO index were evaluated as indicators of winter temperatures available to right whales. Gulf of Maine SST, a time-lagged NAO index, and indices of *C. finmarchicus* abundance were evaluated as proxies of prey availability^63,64^. Daily mean SST data for the Gulf of Maine region (41°N–45.8°N, 71°W–64°W; Fig. 1) were acquired from the NOAA OI SST V2 high resolution (0.25° latitude) dataset (https://www.esrl.noaa.gov/psd). SST data for this region were averaged from April through October for each year, representative of the timeframe prior to right whale migration to the SEUS. An annual summer SST anomaly was calculated as the difference from the mean summer SST for 1989–2015. Monthly mean NAO index values were acquired from the National Weather Service Climate Prediction Center (http://www.cpc.ncep.noaa.gov) and averaged from October through April for each winter.

Abundance data for *C. finmarchicus* were acquired from NOAA EcoMon plankton surveys^55^ and subset for sampling locations in the Gulf of Maine region (north of 41°N and east of 71°W). These surveys consist of bongo tows to depths of 200 m (or 5 m from the ocean floor), with a net mesh size (0.333 mm) known to reliably catch *C. finmarchicus* in life stages that have been associated with foraging right whales (stage C3 and older)^65,66^. Using data from 1990 to 2015 and the *mgcv* package in R^67^, we estimated the climatological seasonal cycle of *C. finmarchicus* by fitting a cyclic regression spline for log-transformed abundance as a function of ordinal date. Daily anomalies were calculated as the difference between this expected abundance and the observed abundance, then averaged across each year to calculate an annual abundance index^55,68^. This process was repeated using a subset of data from the western Gulf of Maine (west of 68°W; Fig. 1) during January-June of each year, and a subset of data from the eastern Gulf of Maine (east of 68°W) during July-December of each year, corresponding with the general, historical patterns of right whale distribution in this region^11^.

Right whale photo-identification and survey data are available through request from the North Atlantic Right Whale Consortium (https://www.narwc.org). EcoMon plankton data are available from NOAA Fisheries Northeast Science Center (https://www.nefsc.noaa.gov/epd/oceanography).

### Statistical modeling

We used multistate capture-recapture models^18,69^ to estimate migration probabilities to the SEUS across demographic groups and years. We defined *S*_*t,j*_ as the probability that an individual alive in year *t* in state *j* survives to year *t * + 1, and ψ_*t,j,k*_ as the probability that an individual transitions to state *k* in year *t*, given that it was in state *j* in year *t* − 1 and survived from *t* − 1 to *t*. We defined *p*_*t,i,j*_ as the probability that an individual alive and in state *j* in year *t* is observed during sampling period *i* in year *t*. Juvenile and adult females (but not males or whales of unknown sex) were permitted to transition to and from the breeder state, but females were not permitted to breed in consecutive years^46,70^ (Fig. 2). Note that our dataset limits inferences to migration to the SMA, rather than the broader SEUS region.

To improve the precision of estimates, we used Pollock’s robust sampling design^24^, consisting of two sampling periods each winter. To permit staggered right whale arrival to, and departure from, the SEUS^47^, we extended the “emigration only” model presented by Kendall^25^, which relaxes the typical closure assumption for robust design models (see Supplementary Information for more details and simulation results; see also Converse, *et al*.^23^). Sightings from each winter were pooled into one of two periods based on the date prior to (or after) which all individuals detected that winter had been observed at least once. Consequently, we assumed that all individuals that had migrated to the SEUS that winter were available for capture at some point during the first period and individuals were permitted to leave the study area before the second period. This period division date varied across winters, likely reflecting variation in residency, but was generally near the end of the winter season (mean = March 6, range = January 29 – April 19).

We used a step-down approach^71^ to evaluate candidate models specified *a priori* for each parameter type (*S*, ψ, and *p*). We first evaluated models for *p* using the most complex candidate model for *S* and ψ, then evaluated models for *S* using the top model for *p* and most complex model for ψ, and finally evaluated models for ψ using the top model for *p* and *S*. Models were fit with Program MARK^72^ through the R package *RMark*^73^ and evaluated using AICc^26^. Parameter counts based on the model structure, rather than those reported by Program MARK, were used to calculate AICc; this method may count unidentifiable parameters in some models but is a more conservative approach, as it will tend to prefer simpler models^73^. To assess goodness of fit and overdispersion (*ĉ*), we applied the JMV goodness of fit tests for multistate models^74^ to the global model for our data (parameters with full variation across years, age, sex, and observable states) in Program U-CARE^75^ (version 2.3.2).

In all models, *p* for individuals in the unobservable state (i.e., not present in the SEUS study area) was fixed to 0, and we assumed no behavioral response to capture (photographing from aircraft). Candidate models for *p* included effects of state, survey effort, interaction effects of state and effort, and additive effects of state and effort, as well as a model where *p* was constant across all states and sampling periods. For models with survey effort as a covariate, effort was calculated as the number of EWS flights in the SMA during the sampling period. We constrained *S* to be equal for all states and constant across time to minimize issues of parameter identifiability^22,76^, because ψ was the primary parameter of interest in this study, and because large variation in *S* is not expected for long-lived mammalian species^41,77^. Two candidate models for *S*, in which *S* was either different or equal between juveniles and adults, were evaluated.

Our candidate models for ψ can be characterized by how they account for variation across demographic groups and for variation across time or with temporal covariates. We tested all combinations of models for demographic groups and time. We considered eight candidate models characterizing variation in ψ across demographic groups: (1) The most complex model included separate intercepts and time effects for each demographic group (juvenile females migrating to the SEUS as breeders, adult females migrating as breeders, juvenile females migrating as non-breeders, adult females migrating as non-breeders, juvenile males, and adult males). Transitions were Markovian, such that they depended on the state an individual was in during the previous winter. (2) The same as model 1, but transitions were non-Markovian and did not differ based on the previous state. This model represents the hypothesis that migration to the SEUS in a given winter is not influenced by whether the individual migrated the previous winter or (in the case of females) the individual’s reproductive state in the previous year. (3) The same as model 1, but individuals were not permitted to transition between an observable and unobservable state. This model represents the hypotheses that temporary emigration does not occur and that partial migration is not condition-dependent (i.e., individuals either always or never migrate to the SEUS). (4) The same as model 1, but no differences between juveniles and adults. (5) Separate intercepts for each demographic group, but time effects were shared (additive) across all groups. This model represents the hypothesis that inter-annual patterns in migration are the same for all demographic groups. (6) Separate intercepts for each demographic group but no differences across sex, and time effects that differed between individuals migrating as breeders and as non-breeders but are otherwise shared. This model represents the hypothesis that temporal patterns in migration depend on an individual’s reproductive state. (7) The same as model 6, but model intercepts included a sex effect. (8) Separate intercepts for each demographic group but no differences between juveniles and adults transitioning from the breeding state, and separate time effects for juveniles migrating as breeders, adults migrating as breeders, juveniles that did not calve in the previous year migrating as non-breeders, adults that did not calve in the previous year migrating as non-breeders, and females that calved in the previous year migrating as non-breeders. Models 6–8 are intermediate between models 1 and 5, where patterns in migration are shared across some, but not all, demographic groups.

We considered eleven candidate models characterizing time effects on ψ that interacted with the eight candidate models characterizing variation across demographic groups: (A) Time-constant, with no variation in migration probability across years. (B) Full time variation, where year was a fixed effect with separate estimates for each of the 21 year-intervals in the study. Some ψ parameters in this model were unidentifiable due to deficient data (*e.g*., ψ from the unobservable state for the first year-interval of the study; ψ for adult males from 1999, during which no adult males were observed); transitions to observable states were fixed to 0 in these cases to reduce their influence on other parameter estimates and model selection^76^. (C) Gulf of Maine SST anomaly from the preceding summer as a covariate. (D) NAO index from the concurrent winter as a covariate. (E) NAO index from two winters prior as a covariate. (F) Gulf of Maine *C. finmarchicus* annual index from the preceding year as a covariate. (G) Gulf of Maine *C. finmarchicus* annual index averaged over the preceding two years as a covariate. (H) Spring (January-June) *C. finmarchicus* annual index for the western Gulf of Maine from the preceding year as a covariate. (I) Spring *C. finmarchicus* annual index for the western Gulf of Maine averaged over the preceding two years as a covariate. (J) Fall (July-December) *C. finmarchicus* annual index for the eastern Gulf of Maine from the preceding year as a covariate. (K) Fall *C. finmarchicus* annual index for the eastern Gulf of Maine averaged over the preceding two years as a covariate.

## Electronic supplementary material

Supplementary Information

## References

[1] Chapman BB (2012). Partial migration in fishes: causes and consequences. J. Fish Biol..

[2] Chapman BB, Brönmark C, Nilsson J-Å, Hansson L-A (2011). The ecology and evolution of partial migration. Oikos.

[3] Lundberg P (1988). The evolution of partial migration in birds. Trends Ecol. Evol..

[4] Lockyer C (1984). Review of baleen whale (Mysticeti) reproduction and implications for management. Report International Whaling Commission.

[5] Clapham P (2001). Why do Baleen Whales Migrate?: A Response to Corkeron and Connor. Mar. Mammal Sci..

[6] Corkeron PJ, Connor RC (1999). Why do baleen whales migrate?. Mar. Mammal Sci..

[7] Geijer CKA, Notarbartolo di Sciara G, Panigada S (2016). Mysticete migration revisited: are Mediterranean fin whales an anomaly?. Mammal Rev..

[8] Davis GE (2017). Long-term passive acoustic recordings track the changing distribution of North Atlantic right whales (*Eubalaena glacialis*) from 2004 to 2014. Sci. Rep..

[9] Kraus, S. D. & Rolland, R. *The urban whale: North Atlantic right whales at the crossroads*. (Harvard University Press, 2007).

[10] Cole T (2013). Evidence of a North Atlantic right whale *Eubalaena glacialis* mating ground. Endanger. Species Res..

[11] Schick RS (2013). Using hierarchical Bayes to understand movement, health, and survival in the endangered North Atlantic right whale. PLoS ONE.

[12] Hamilton, P. K., Knowlton, A. R. & Marx, M. K. Right whales tell their own stories: the photo identification catalog. In *The urban whale: North Atlantic right whales at the crossroads* 75–104 (Harvard University Press, 2007).

[13] Hamilton PK, Cooper LA (2010). Changes in North Atlantic right whale (*Eubalaena glacialis*) cow-calf association times and use of the calving ground: 1993-2005. Mar. Mammal Sci..

[14] Waring, G. T., Josephson, E., Maze-Foley, K. & Rosel, P. E. US Atlantic and Gulf of Mexico Marine Mammal Stock Assessments – 2015. *NOAA Technical Memorandum NMFS-NE* **238** (2016).

[15] Pettis, H. M. & Hamilton, P. K. North Atlantic Right Whale Consortium Annual Report Card. *Report to the North Atlantic Right Whale Consortium, November 2016* (2016).

[16] Meyer-Gutbrod E, Greene C, Sullivan P, Pershing A (2015). Climate-associated changes in prey availability drive reproductive dynamics of the North Atlantic right whale population. Mar. Ecol. Prog. Ser..

[17] Pendleton D (2009). Regional-scale mean copepod concentration indicates relative abundance of North Atlantic right whales. Mar. Ecol. Prog. Ser..

[18] Hestbeck JB, Nichols JD, Malecki RA (1991). Estimates of movement and site fidelity using mark-resight data of wintering Canada Geese. Ecology.

[19] Lebreton JD, Pradel R (2002). Multistate recapture models: modelling incomplete individual histories. J. Appl. Stat..

[20] Kendall WL, Nichols JD (2002). Estimating state-transition probabilities for unobservable states using capture-recapture/resighting data. Ecology.

[21] Kendall WL, Nichols JD, Hines JE (1997). Estimating temporary emigration using capture-recapture data with Pollock’s robust design. Ecology.

[22] Schaub M, Gimenez O, Schmidt BR, Pradel R (2004). Estimating survival and temporary emigration in the multistate capture–recapture framework. Ecology.

[23] Converse S, Kendall W, Doherty P, Ryan P (2009). Multistate models for estimation of survival and reproduction in Grey-headed Albatross *(Thalassarche chrysostoma)*. The Auk.

[24] Pollock KH (1982). A capture-recapture design robust to unequal probability of capture. J. Wildlife Manage..

[25] Kendall WL (1999). Robustness of closed capture-recapture methods to violations of the closure assumption. Ecology.

[26] Burnham, K. P., Anderson, D. R. & Burnham, K. P. *Model selection and multimodel inference: a practical information-theoretic approach*. (Springer, 2002).

[27] Solow AR, Bjorndal KA, Bolten AB (2002). Annual variation in nesting numbers of marine turtles: the effect of sea surface temperature on re-migration intervals. Ecol. Letters.

[28] Brodersen J, Nilsson PA, Hansson L-A, Skov C, Brönmark C (2008). Condition-dependent individual decision-making determines cyprinid partial migration. Ecology.

[29] Rolland R (2016). Health of North Atlantic right whales *Eubalaena glacialis* over three decades: from individual health to demographic and population health trends. Mar. Ecol. Prog. Ser..

[30] van der Hoop J, Corkeron P, Moore M (2017). Entanglement is a costly life-history stage in large whales. Ecol. Evol..

[31] Jahn AE, Levey DJ, Hostetler JA, Mamani AM (2010). Determinants of partial bird migration in the Amazon Basin. J. Anim. Ecol..

[32] Brodie PF (1975). Cetacean energetics, an overview of intraspecific size variation. Ecology.

[33] Ryg M (1993). Scaling of insulation in seals and whales. J. Zool..

[34] Skov C (2011). Sizing up your enemy: individual predation vulnerability predicts migratory probability. P. Roy. Soc. B-Biol. Sci..

[35] Craig AS, Herman LM (1997). Sex differences in site fidelity and migration of humpback whales (*Megaptera novaeangliae*) to the Hawaiian Islands. Can. J. Zool..

[36] Shaw AK, Levin SA (2011). To breed or not to breed: a model of partial migration. Oikos.

[37] Sironi, M. Behavior and social development of juvenile southern right whales (*Eubalaena australis*) and interspecific interactions at Península Valdés, Argentina. (University of Wisconsin-Madison, Madison, 2004).

[38] Parks SE (2007). Occurrence, composition, and potential functions of North Atlantic right whale (*Eubalaena glacialis*) surface active groups. Mar. Mammal Sci..

[39] Brodersen J, Ådahl E, Brönmark C, Hansson L-A (2008). Ecosystem effects of partial fish migration in lakes. Oikos.

[40] Grieve BD, Hare JA, Saba VS (2017). Projecting the effects of climate change on *Calanus finmarchicus* distribution within the U.S. Northeast Continental Shelf. Sci. Rep..

[41] Pace RM, Corkeron PJ, Kraus SD (2017). State-space mark-recapture estimates reveal a recent decline in abundance of North Atlantic right whales. Ecol. Evol..

[42] Meyer-Gutbrod EL, Greene CH (2018). Uncertain recovery of the North Atlantic right whale in a changing ocean. Glob Change Biol..

[43] Peñaloza CL, Kendall WL, Langtimm CA (2014). Reducing bias in survival under nonrandom temporary emigration. Ecol. Appl..

[44] Carroll EL (2013). Accounting for female reproductive cycles in a superpopulation capture–recapture framework. Ecol. Appl..

[45] Baker C, Perry A, Herman L (1987). Reproductive histories of female humpback whales *Megaptera novaeangliae* in the North Pacific. Mar. Ecol. Prog. Ser..

[46] Davidson, A. R., Rayment, W., Dawson, S. M., Webster, T. & Slooten, E. Estimated calving interval for the New Zealand southern right whale *(Eubalaena australi*s). *New Zeal. J. Mar. Fresh*., 10.1080/00288330.2017.1397034 (2017).

[47] Krzystan AM (2018). Characterizing residence patterns of North Atlantic right whales in the southeastern USA with a multistate open robust design model. Endanger. Species Res..

[48] Patrician MR (2009). Evidence of a North Atlantic right whale calf (*Eubalaena glacialis*) born in northeastern U.S. waters. Mar. Mammal Sci..

[49] McLellan, W. A. *et al*. Winter right whale sightings from aerial surveys of the coastal waters of the US mid-Atlantic. in *15th Biennial Conference on the Biology of Marine Mammals* (2003).

[50] Kendall WL, Hines JE, Nichols JD (2003). Adjusting multistate capture–recapture models for misclassification bias: manatee breeding proportions. Ecology.

[51] Rolland RM, Hunt KE, Kraus SD, Wasser SK (2005). Assessing reproductive status of right whales (*Eubalaena glacialis*) using fecal hormone metabolites. Gen. Comp. Endocr..

[52] Hunt KE, Rolland RM, Kraus SD (2014). Detection of steroid and thyroid hormones *via* immunoassay of North Atlantic right whale (*Eubalaena glacialis*) respiratory vapor. Mar. Mammal Sci..

[53] Mucientes GR, Queiroz N, Sousa LL, Tarroso P, Sims DW (2009). Sexual segregation of pelagic sharks and the potential threat from fisheries. Biol. Letters.

[54] Hazen EL (2017). WhaleWatch: a dynamic management tool for predicting blue whale density in the California Current. J. Appl. Ecol..

[55] Kane J (2007). Zooplankton abundance trends on Georges Bank, 1977–2004. ICES J. Mar. Sci..

[56] Brown, M., Kraus, S., Slay, C. & Garrison, L. Surveying for discovery, science and management. in *The urban whale: North Atlantic right whales at the crossroads* 105–137 (Harvard University Press, 2007).

[57] Keller CA (2006). North Atlantic right whale distribution in relation to sea-surface temperature in the southeastern United States calving grounds. Mar. Mammal Sci..

[58] Gowan TA, Ortega-Ortiz JG (2014). Wintering habitat model for the North Atlantic right whale (*Eubalaena glacialis*) in the southeastern United States. PLoS ONE.

[59] Right W Consortium. North Atlantic Right Whale Consortium IdentificationDatabase. (Accessed: November 2016).

[60] Brown MW, Kraus SD, Gaskin DE, White BN (1994). Sexual composition and analysis of reproductive females in the North Atlantic right whale, *Eubalaena glacialis*, population. Mar. Mammal Sci..

[61] Hamilton P, Knowlton A, Marx M, Kraus S (1998). Age structure and longevity in North Atlantic right whales *Eubalaena glacialis* and their relation to reproduction. Mar. Ecol. Prog. Ser..

[62] Robbins J, Knowlton AR, Landry S (2015). Apparent survival of North Atlantic right whales after entanglement in fishing gear. Biol. Conserv..

[63] Greene C (2000). The response of *Calanus finmarchicus* populations to climate variability in the Northwest Atlantic: basin-scale forcing associated with the North Atlantic Oscillation. ICES J. Mar. Sci..

[64] Greene CH, Pershing AJ (2004). Climate and the conservation biology of North Atlantic right whales: the right whale at the wrong time?. Front. Ecol. Environ..

[65] Anderson JT, Warren WG (1991). Comparison of catch rates among small and large bongo sampler for *Calanus finmarchicus* copepodite stages. Can. J. Fish. Aquat. Sci..

[66] Baumgartner M, Cole T, Campbell R, Teegarden G, Durbin E (2003). Associations between North Atlantic right whales and their prey, *Calanus finmarchicus*, over diel and tidal time scales. Mar. Ecol. Prog. Ser..

[67] Wood S (2001). N. mgcv: GAMs and generalized ridge regression for R. R News.

[68] Pershing A (2005). Interdecadal variability in the Gulf of Maine zooplankton community, with potential impacts on fish recruitment. ICES J. Mar. Sci..

[69] Arnason NA (1973). The estimation of population size, migration rates and survival in a stratified population. Researches on Population Ecology.

[70] Knowlton AR, Kraus SD, Kenney RD (1994). Reproduction in North Atlantic right whales (*Eubalaena glacialis*). Can. J. Zool..

[71] Lebreton J-D, Burnham KP, Clobert J, Anderson DR (1992). Modeling survival and testing biological hypotheses using marked animals: A unified approach with case studies. Ecol. Monogr..

[72] White GC, Burnham KP (1999). Program MARK: survival estimation from populations of marked animals. Bird Study.

[73] Laake, J. L. RMark: An R interface for analysis of capture-recapture data with MARK. AFSC Processed Rep 2013-01. Alaska Fisheries Science Center, NOAA, National Marine Fisheries Service. (2013).

[74] Pradel R, Wintrebert CMA, Gimenez O (2003). A proposal for a goodness-of-fit test to the Arnason-Schwarz multisite capture-recapture model. Biometrics.

[75] Choquet R, Lebreton J-D, Gimenez O, Reboulet A-M, Pradel R (2009). U-CARE: Utilities for performing goodness of fit tests and manipulating CApture–REcapture data. Ecography.

[76] Bailey LL, Converse SJ, Kendall WL (2010). Bias, precision, and parameter redundancy in complex multistate models with unobservable states. Ecology.

[77] Gaillard J-M, Yoccoz NG (2003). Temporal variation in survival of mammals: a case of environmental canalization?. Ecology.

