# Supplementary material for: Temporal and demographic variation in partial migration of the North Atlantic right whale
Source: Sci Rep. 2019 Jan 23;9:353. doi: 10.1038/s41598-018-36723-3 (PMC6344554; doi:10.1038/s41598-018-36723-3)
Supplement: Supplementary file 1 — Supplementary Information [file 41598_2018_36723_MOESM1_ESM.pdf]

Supplementary Information

Temporal and demographic variation in partial migration of the North Atlantic right whale

Timothy A. Gowan[1,2*], Joel G. Ortega-Ortiz[3], Jeffrey A. Hostetler[1], Philip K. Hamilton[4], Amy R. Knowlton[4], Katharine A. Jackson[1], R. Clay George[5], Cynthia R. Taylor[6], Patricia J. Naessig[6]

[1] Fish and Wildlife Research Institute, Florida Fish and Wildlife Conservation Commission, St. Petersburg, Florida 33701, USA
[2] Department of Wildlife Ecology and Conservation, University of Florida, Gainesville, Florida 32611, USA
[3] Cooperative Institute for Marine and Atmospheric Studies, Rosenstiel School of Marine and Atmospheric Science, University of Miami, Miami, Florida 33149, USA
[4] Anderson Cabot Center for Ocean Life, New England Aquarium, Boston, Massachusetts 02110, USA
[5] Georgia Department of Natural Resources, Wildlife Conservation Section, Brunswick, Georgia 31520, USA
[6] Sea to Shore Alliance, Sarasota, Florida 34233, USA
[*] Corresponding author: tim.gowan@myfwc.com

Supplement S1

Multistate capture-recapture models that include an unobservable state have been used to estimate survival and temporary emigration probabilities. However, some parameters in these models may not be identifiable when there is only one sampling occasion during each primary period [20, 22]. Even when constraints are imposed to reduce model complexity, parameter estimates can still have poor precision or be biased due to finite sample sizes (Table S1). A robust sampling design [21, 24] with multiple secondary sampling occasions during each primary period can improve parameter estimability (Table S2).

The robust design, however, typically requires a closure assumption (i.e., all individuals in an observable state are present and available for capture during all sampling occasions within a primary period) that is often difficult to achieve in practice. For example, right whales appear to have staggered arrival to and departure from their southeastern U.S. wintering grounds [47]. Kendall [25] presented an “emigration only” model that relaxes this closure assumption by requiring all observable individuals to be present and available for capture on the first sampling occasion but permits them to become unavailable (*e.g.*, leave the study area) before sampling ends (on subsequent sampling occasions). All sampling occasions after the first are pooled together, resulting in two sampling occasions per primary period for analysis.

In addition to allowing emigration before sampling ends, we extended Kendall's model to allow individuals to arrive after the first sampling occasion and to have multiple arrivals and departures within a primary period. In our model, we assume that for a primary period with T total sampling occasions (*e.g.*, survey days), no new individuals arrive after sampling occasion t_a, where $t_a < T$. Data are pooled for analysis across occasions 1 through t_a and $t_a + 1$ through T, resulting in two sampling occasions per primary period with the same assumptions as Kendall's emigration only model: one occasion where all individuals are available for capture and another where some individuals may have become unavailable. Performance of this model (Table S3) was comparable to that of the robust design model requiring full closure (Table S2). Note that this closure violation reduces the effective capture probabilities, and the level of reduction is determined by the underlying arrival and departure probabilities.

parameter	true value	mean estimate	CV	rRMSE	coverage
p	0.70	0.72	0.22	0.23	**0.82**
$\psi_{2,A,X}$	0.80	0.80	0.10	0.10	0.94
$\psi_{3,A,X}$	0.60	0.60	0.26	0.25	0.95
$\psi_{4,A,X}$	0.40	0.39	0.40	0.39	0.86
$\psi_{5,A,X}$	0.20	0.19	0.84	0.82	**0.76**
$\psi_{6,A,X}$	0.40	0.39	0.35	0.34	**0.80**
$\psi_{7,A,X}$	0.60	0.60	0.17	0.16	**0.84**
$\psi_{8,A,X}$	0.80	0.80	0.08	0.08	0.91
$\psi_{2,X,A}$	0.20	0.50	0.00	1.50	**0.47**
$\psi_{3,X,A}$	0.40	0.41	0.34	0.35	0.92
$\psi_{4,X,A}$	0.60	0.59	0.31	0.31	0.85
$\psi_{5,X,A}$	0.80	0.75	0.32	0.30	**0.77**
$\psi_{6,X,A}$	0.60	0.51	0.73	0.63	**0.68**
$\psi_{7,X,A}$	0.40	0.39	0.46	0.45	0.89
$\psi_{8,X,A}$	0.20	0.20	0.37	0.37	0.94
S	0.90	0.90	0.03	0.03	0.95

Table S1. Results of multistate model with one sampling occasion per primary period from 1,000 simulated datasets. Datasets were based on a Jolly-Seber model with an initial population size of 100 individuals, 8 primary periods each with a constant recruitment probability of 0.3, and capture probability (p = 0.7) and survival probability (S = 0.9) constant across time. Transition probability from observable to unobservable state ($\psi_{t,A,X}$) and transition probability from unobservable to observable state ($\psi_{t,X,A}$) varied across time. Model metrics include the mean, coefficient of variation (CV), root mean square error normalized by the true parameter value

(rRMSE), and coverage (from 95% confidence limits) for parameter estimates across all simulations. Coverage values < 0.85 in bold; transition probability from unobservable to observable state during the first time interval ($\psi_{2,X,A}$) is intrinsically unidentifiable.

parameter	true value	mean estimate	CV	rRMSE	coverage
p	0.33	0.33	0.04	0.04	0.96
$\psi_{2,A,X}$	0.80	0.80	0.08	0.08	0.96
$\psi_{3,A,X}$	0.60	0.60	0.21	0.21	0.97
$\psi_{4,A,X}$	0.40	0.40	0.27	0.27	0.96
$\psi_{5,A,X}$	0.20	0.20	0.44	0.44	0.95
$\psi_{6,A,X}$	0.40	0.40	0.18	0.18	0.97
$\psi_{7,A,X}$	0.60	0.60	0.11	0.11	0.96
$\psi_{8,A,X}$	0.80	0.80	0.06	0.06	0.96
$\psi_{2,X,A}$	0.20	0.50	0.00	1.50	**0.46**
$\psi_{3,X,A}$	0.40	0.40	0.26	0.26	0.97
$\psi_{4,X,A}$	0.60	0.60	0.22	0.23	0.97
$\psi_{5,X,A}$	0.80	0.78	0.22	0.21	0.92
$\psi_{6,X,A}$	0.60	0.59	0.48	0.48	0.96
$\psi_{7,X,A}$	0.40	0.41	0.35	0.35	0.99
$\psi_{8,X,A}$	0.20	0.20	0.32	0.33	0.97
S	0.90	0.90	0.03	0.03	0.96

Table S2. Results of multistate model with multiple sampling occasions per closed primary period from 1,000 simulated datasets. Datasets were based on a Jolly-Seber model with an initial population size of 100 individuals, 8 primary periods each with 3 secondary sampling occasions and a constant recruitment probability of 0.3, and capture probability (p = 0.33 for each secondary occasion) and survival probability (S = 0.9) constant across time. Transition probability from observable to unobservable state ($\psi_{t,A,X}$) and transition probability from unobservable to observable state ($\psi_{t,X,A}$) varied across time. Model metrics are same as in Table S1. Coverage values < 0.85 in bold; transition probability from unobservable to observable state during the first time interval ($\psi_{2,X,A}$) is intrinsically unidentifiable.

parameter	true value	mean estimate	CV	rRMSE	coverage
p_1	NA	0.82	0.04	NA	NA
p_2	NA	0.17	0.08	NA	NA
$\psi_{2,A,X}$	0.80	0.80	0.07	0.07	0.95
$\psi_{3,A,X}$	0.60	0.60	0.16	0.16	0.95
$\psi_{4,A,X}$	0.40	0.40	0.20	0.20	0.96
$\psi_{5,A,X}$	0.20	0.20	0.38	0.37	0.93
$\psi_{6,A,X}$	0.40	0.40	0.18	0.18	0.93
$\psi_{7,A,X}$	0.60	0.60	0.09	0.09	0.95
$\psi_{8,A,X}$	0.80	0.80	0.05	0.05	0.95
$\psi_{2,X,A}$	0.20	0.50	0.00	1.50	**0.45**
$\psi_{3,X,A}$	0.40	0.40	0.20	0.20	0.96
$\psi_{4,X,A}$	0.60	0.59	0.17	0.17	0.96
$\psi_{5,X,A}$	0.80	0.80	0.15	0.15	0.93
$\psi_{6,X,A}$	0.60	0.61	0.38	0.38	0.93
$\psi_{7,X,A}$	0.40	0.42	0.32	0.33	0.96
$\psi_{8,X,A}$	0.20	0.21	0.33	0.34	0.95
S	0.90	0.90	0.02	0.02	0.93

Table S3. Results of multistate model with multiple sampling occasions per open primary period from 1,000 simulated datasets. Datasets were based on a Jolly-Seber model with an initial population size of 100 individuals, 8 primary periods each with 6 secondary sampling occasions and a constant recruitment probability of 0.3, and capture probability (p = 0.7 for each secondary occasion) and survival probability (S = 0.9) constant across time. Transition probability from observable to unobservable state ($\psi_{t,A,X}$) and transition probability from unobservable to observable state ($\psi_{t,X,A}$) varied across time. Additional parameters (not estimated by the model) for each open primary period were constant across primary periods and as follows: arrival (and re-entry) probabilities prior to each secondary sampling occasion (a_1 = 0.1, a_2 = 0.2, a_3 = 0.3, a_4 = 0.4, a_5 = 0, a_6 = 0) and departure probabilities for each secondary occasion interval ($d_{1,2}$ = 0.1, $d_{2,3}$ = 0.2, $d_{3,4}$ = 0.3, $d_{4,5}$ = 0.7, $d_{5,6}$ = 0.9). No arrivals occur in secondary occasions 5 or 6; data were pooled for analysis across occasions 1 through 4 into p_1, and 5 through 6 into p_2. Model metrics are same as in Table S1. Coverage values < 0.85 in bold; transition probability from unobservable to observable state during the first time interval ($\psi_{2,X,A}$) is intrinsically unidentifiable.

Supplement S2

Year	Non-calving Adult Females	Adult Males/Unk	Calving Females (≥ age 9)	Calving Females (< age 9)	Non-calving Juvenile Females	Juvenile Males/Unk
1994	2	1	6	0	7	5
1995	0	1	6	0	3	2
1996	6	15	18	0	13	17
1997	4	0	14	1	6	3
1998	8	7	5	0	6	9
1999	4	0	3	0	1	2
2000	3	13	1	0	6	1
2001	15	13	29	0	0	2
2002	10	1	16	0	10	5
2003	8	12	18	0	7	9
2004	7	9	13	1	13	17
2005	13	40	25	0	27	38
2006	5	14	18	0	25	31
2007	4	17	17	1	21	45
2008	8	44	17	4	31	57
2009	8	48	31	6	37	66
2010	5	50	18	0	51	68
2011	6	19	16	1	42	32
2012	3	2	6	0	19	20
2013	3	1	15	4	7	5
2014	6	7	10	0	5	3
2015	2	2	16	0	1	1

Table S4. Number of individual North Atlantic right whales identified in the southeastern U.S. seasonal management area by Early Warning System aerial surveys by demographic group, 1994-2015.

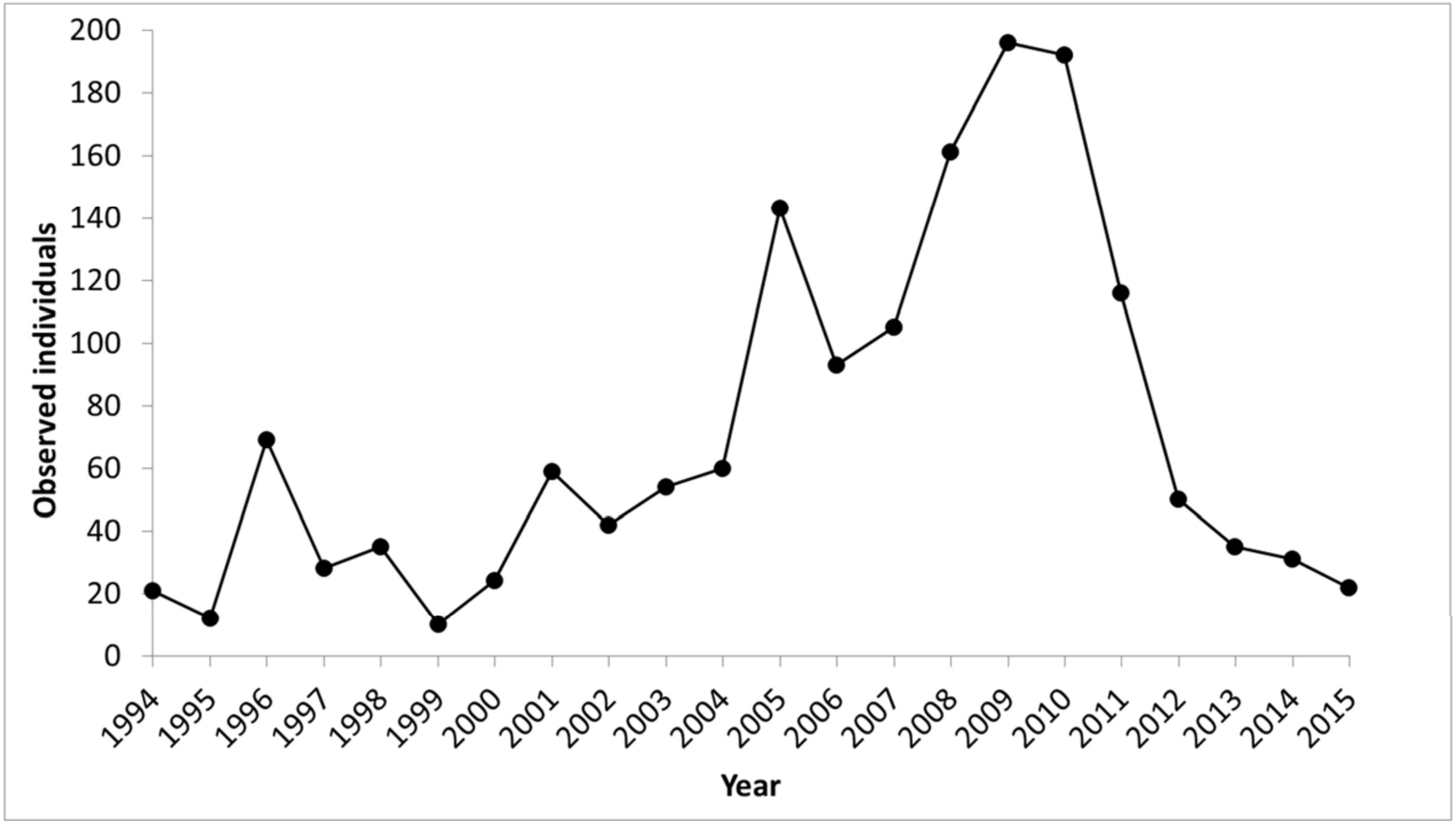

Figure S1. Number of individual North Atlantic right whales identified in the southeastern U.S. seasonal management area by Early Warning System aerial surveys, 1994-2015. Excludes first-year calves.

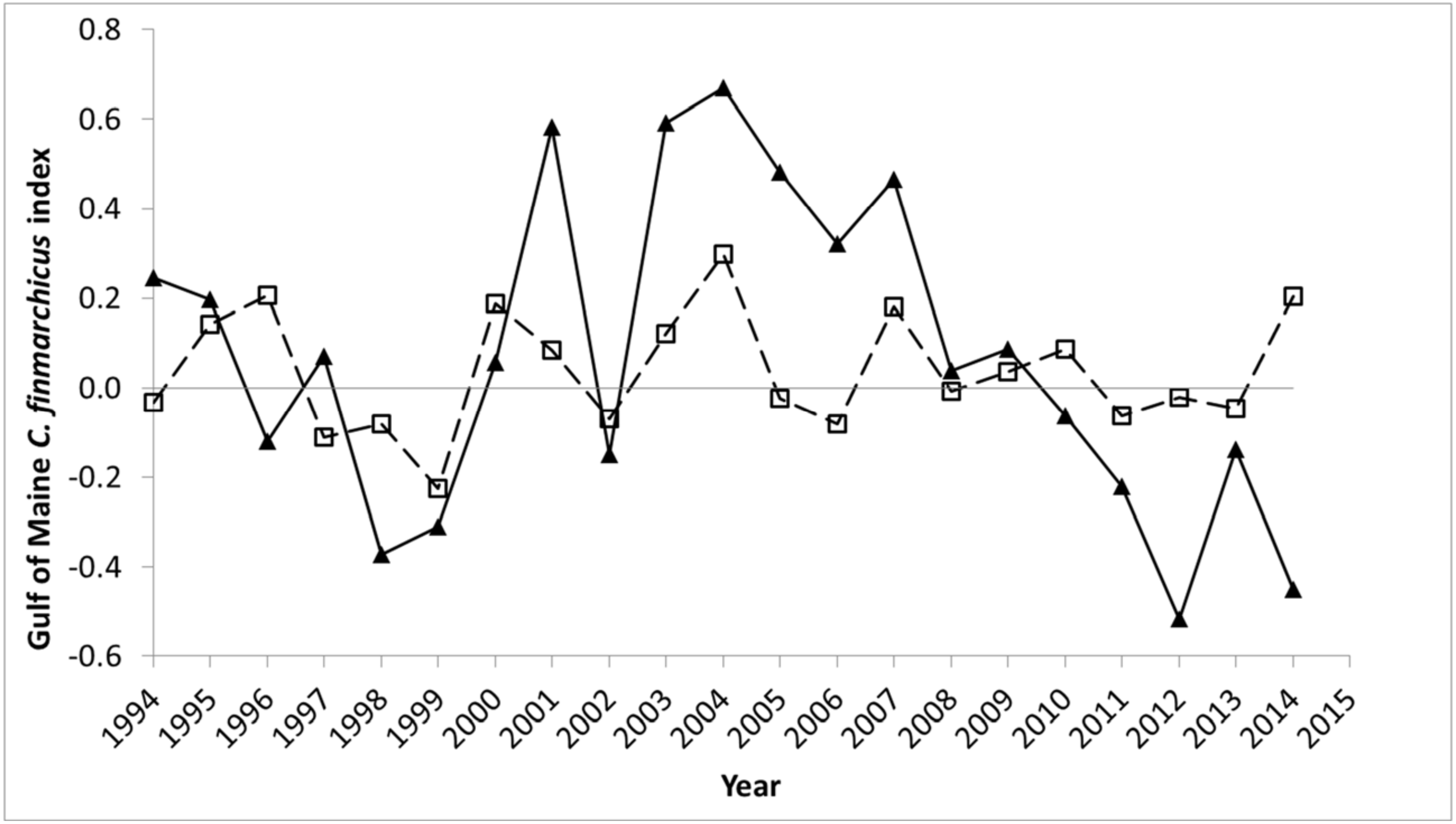

Figure S2. Gulf of Maine annual *C. finmarchicus* anomaly for spring (January – June) in the western Gulf of Maine (west of 68°W; open squares, dashed line) and for fall (July – December) in the eastern Gulf of Maine (east of 68°W; closed triangles, solid line). Data from NOAA EcoMon plankton surveys.

Supplement S3

Table S5. Model selection results for capture (*p*), survival (*S*), and state transition (ψ) probabilities. Models with the lowest AICc (Akaike's Information Criterion corrected for small sample sizes) values were deemed the best model in each step. Models for *p* include interaction effects of state and survey effort, additive effects of state and effort, effect of survey effort, effect of state, and no effects (.). Models for *S* include no effects and an effect of age-class (juveniles different than adults). Models for ψ include eight different models for demographic effects that interact with eleven time effects. Demographic models (see text for more details) include: separate intercepts and time effects for each demographic group (1); non-Markovian transitions (2); no temporary emigration (3); no differences between juveniles and adults (4); separate intercepts, but time effects additive across all groups (5); separate intercepts but no differences across sex, and time effects additive within reproductive state (6); separate intercepts, and time effects additive within reproductive state (7); and separate intercepts but no differences between juveniles and adults transitioning from the breeding state, and time effects additive within reproductive state and age-class (8). Time effects include: no variation across time (.); full time variation (time); summer Gulf of Maine sea surface temperature anomaly (GoM_SST); North Atlantic Oscillation index from the concurrent winter (NAO); North Atlantic Oscillation index from two winters prior (NAO_lag2); Gulf of Maine *Calanus finmarchicus* annual index (CAL); Gulf of Maine *C. finmarchicus* annual index averaged over the preceding two years (CAL_2avg); western Gulf of Maine *C. finmarchicus* spring index (WCAL); western Gulf of Maine *C. finmarchicus* spring index averaged over the preceding two years (WCAL_2avg); eastern Gulf of Maine *C. finmarchicus* fall index (ECAL); and eastern Gulf of Maine *C. finmarchicus* fall index averaged over the preceding two years (ECAL_2avg).

Step 1: *p*

S	ψ	*p*	Parameters	Deviance	AICc	ΔAICc
ageclass	1.time	state*effort	281	4933.4	5611.2	0.0
ageclass	1.time	state+effort	280	4970.6	5645.5	34.3
ageclass	1.time	effort	279	5031.6	5703.6	92.4
ageclass	1.time	state	279	7310.5	7982.5	2371.3
ageclass	1.time	.	278	7359.8	8028.8	2417.7

Step 2: *S*

S	ψ	*p*	Parameters	Deviance	AICc	ΔAICc
.	1.time	state*effort	280	4932.2	5607.1	0.0
ageclass	1.time	state*effort	281	4933.4	5611.2	4.1

Step 3: ψ

S	ψ	*p*	Parameters	Deviance	AICc	ΔAICc
.	8.time	state*effort	120	5140.3	5399.3	0.0
.	5.time	state*effort	40	5332.9	5414.9	15.6
.	7.time	state*effort	53	5310.4	5420.0	20.7
.	6.time	state*effort	52	5335.3	5442.8	43.5
.	2.time	state*effort	132	5230.8	5517.9	118.6
.	1.ECAL_2avg	state*effort	33	5521.0	5588.4	189.1
.	5.ECAL_2avg	state*effort	20	5563.1	5603.6	204.3
.	1.NAO	state*effort	33	5537.3	5604.7	205.4
.	1.time	state*effort	280	4932.2	5607.1	207.8
.	1.CAL_2avg	state*effort	33	5540.6	5608.0	208.7
.	5.NAO	state*effort	20	5568.4	5608.9	209.6
.	8.NAO	state*effort	23	5562.5	5609.2	209.9
.	5.WCAL	state*effort	20	5571.4	5611.9	212.6
.	8.ECAL_2avg	state*effort	23	5567.6	5614.2	214.9
.	5.WCAL_2avg	state*effort	20	5575.3	5615.8	216.5
.	1.GoM_SST	state*effort	33	5553.4	5620.8	221.5
.	5.CAL_2avg	state*effort	20	5585.6	5626.1	226.8
.	1.CAL	state*effort	33	5559.3	5626.7	227.4
.	1.WCAL_2avg	state*effort	33	5559.7	5627.0	227.7
.	4.time	state*effort	152	5293.5	5628.5	229.2
.	1.WCAL	state*effort	33	5561.8	5629.2	229.9
.	8.WCAL_2avg	state*effort	23	5582.8	5629.5	230.2
.	5.ECAL	state*effort	20	5597.2	5637.7	238.4
.	8.CAL_2avg	state*effort	23	5593.7	5640.4	241.1
.	7.ECAL_2avg	state*effort	15	5611.3	5641.6	242.3
.	7.NAO	state*effort	15	5615.1	5645.4	246.1
.	8.ECAL	state*effort	23	5599.6	5646.3	247.0
.	5.CAL	state*effort	20	5606.5	5647.0	247.7
.	5.GoM_SST	state*effort	20	5609.2	5649.8	250.5
.	7.WCAL_2avg	state*effort	15	5620.9	5651.2	251.9
.	7.WCAL	state*effort	15	5621.8	5652.1	252.8
.	8.CAL	state*effort	23	5612.7	5659.4	260.1
.	1. .	state*effort	19	5623.8	5662.3	263.0
.	5. .	state*effort	19	5623.8	5662.3	263.0

.	7.CAL_2avg	state*effort	15	5632.9	5663.2	263.9
.	1.NAO_lag2	state*effort	33	5596.0	5663.4	264.1
.	5.NAO_lag2	state*effort	20	5623.8	5664.3	265.0
.	6.ECAL_2avg	state*effort	14	5643.1	5671.4	272.1
.	6.NAO	state*effort	14	5648.9	5677.2	277.9
.	7.ECAL	state*effort	15	5648.0	5678.3	279.0
.	1.ECAL	state*effort	33	5611.4	5678.8	279.5
.	6.WCAL_2avg	state*effort	14	5656.8	5685.0	285.7
.	6.WCAL	state*effort	14	5658.1	5686.4	287.1
.	7.CAL	state*effort	15	5656.7	5687.0	287.7
.	7.NAO_lag2	state*effort	15	5660.0	5690.3	291.0
.	7.GoM_SST	state*effort	15	5661.2	5691.5	292.2
.	6.CAL_2avg	state*effort	14	5667.5	5695.8	296.5
.	7. .	state*effort	13	5673.5	5699.8	300.5
.	6.ECAL	state*effort	14	5682.9	5711.2	311.9
.	6.CAL	state*effort	14	5693.3	5721.6	322.3
.	6.GoM_SST	state*effort	14	5696.9	5725.1	325.8
.	6.NAO_lag2	state*effort	14	5698.9	5727.1	327.8
.	6. .	state*effort	12	5711.0	5735.2	335.9
.	2.ECAL_2avg	state*effort	17	5737.5	5771.9	372.6
.	2.NAO	state*effort	17	5740.5	5774.9	375.6
.	8.WCAL	state*effort	23	5729.8	5776.5	377.2
.	2.WCAL_2avg	state*effort	17	5763.9	5798.3	399.0
.	8.NAO_lag2	state*effort	23	5752.3	5799.0	399.7
.	8. .	state*effort	18	5769.7	5806.1	406.8
.	2.CAL_2avg	state*effort	17	5772.2	5806.6	407.3
.	2.WCAL	state*effort	17	5772.6	5807.0	407.7
.	8.GoM_SST	state*effort	23	5761.6	5808.3	409.0
.	4.ECAL_2avg	state*effort	19	5776.2	5814.6	415.3
.	4.NAO	state*effort	19	5787.1	5825.6	426.3
.	2.ECAL	state*effort	17	5792.3	5826.6	427.3
.	4.CAL_2avg	state*effort	19	5789.1	5827.6	428.3
.	2.GoM_SST	state*effort	17	5795.6	5830.0	430.7
.	2.NAO_lag2	state*effort	17	5802.1	5836.5	437.2
.	2.CAL	state*effort	17	5802.7	5837.1	437.8
.	4.ECAL	state*effort	19	5801.1	5839.6	440.3
.	4.CAL	state*effort	19	5802.0	5840.4	441.1
.	2. .	state*effort	11	5819.6	5841.8	442.5
.	4.GoM_SST	state*effort	19	5803.5	5842.0	442.7
.	4.WCAL	state*effort	19	5804.3	5842.8	443.5
.	4.WCAL_2avg	state*effort	19	5809.6	5848.1	448.8
.	4.NAO_lag2	state*effort	19	5835.8	5874.2	474.9
.	4. .	state*effort	12	5862.9	5887.1	487.8

.	3.time	state*effort	47	6491.5	6588.3	1189.0
.	3.ECAL_2avg	state*effort	9	6595.0	6613.2	1213.9
.	3.CAL_2avg	state*effort	9	6600.5	6618.6	1219.3
.	3.WCAL_2avg	state*effort	9	6601.6	6619.7	1220.4
.	3.WCAL	state*effort	9	6603.1	6621.2	1221.9
.	3.ECAL	state*effort	9	6608.2	6626.3	1227.0
.	3.NAO	state*effort	9	6609.1	6627.2	1227.9
.	3.CAL	state*effort	9	6613.1	6631.2	1231.9
.	3.NAO_lag2	state*effort	9	6620.3	6638.4	1239.1
.	3. .	state*effort	7	6627.0	6641.1	1241.8
.	3.GoM_SST	state*effort	9	6623.8	6641.9	1242.6
